# Radioiodine sinus uptake related to mucosal thickening or aspergilloma: a case series of an unrecognized event well evidenced by SPECT/CT

**DOI:** 10.1186/s40644-016-0105-1

**Published:** 2017-01-13

**Authors:** Renaud Ciappuccini, David Blanchard, Jean-Pierre Rame, Dominique de Raucourt, Emmanuel Babin, Stéphane Bardet

**Affiliations:** 1 Department of Nuclear Medicine and Thyroid Unit, François Baclesse Cancer Centre, 3 Avenue Général Harris - BP 5026, F-14076 CAEN Cedex 05, France; 2 INSERM U1086 “Cancers et Préventions”, François Baclesse Cancer Centre, Caen, France; 3 Department of Head and Neck Surgery, François Baclesse Cancer Centre, Caen, France; 4 Department of Head and Neck Surgery, University Hospital, Caen, France

**Keywords:** Differentiated thyroid cancer, Radioiodine, SPECT/CT, Sinus, Aspergilloma, Case report

## Abstract

**Background:**

False-positive radioiodine (RAI) uptake related to chronic sinusitis and mucocele has only rarely been reported in patients with differentiated thyroid cancer (DTC) even with the recent use of single photon emission tomography with computed tomography (SPECT/CT) acquisition. No other etiology of sinus RAI uptake has been mentioned to date.

**Objectives:**

We report five cases of DTC patients with sinus RAI uptake on post-RAI scintigraphy. SPECT/CT clearly localized RAI uptake either in the sphenoid, the maxillary or the frontal sinus and highly suspected mucosal thickening in four patients and sinus aspergilloma in one patient.

**Conclusion:**

These data confirm the possibility of false-positive sinus RAI uptake, provide a new cause of such benign uptake, i.e. sinus aspergilloma, and demonstrate the clinical relevance of head and neck SPECT/CT acquisition in the diagnosis of such uptake. Nuclear medicine physicians should be aware of this pitfall when interpreting post-RAI scintigraphy.

## Background

False-positive radioiodine (RAI) uptake in chronic frontal sinusitis was reported once in a patient with differentiated thyroid cancer (DTC) [1] and in mucocele [2] twenty years ago using planar imaging. Although single photon emission tomography with computed tomography (SPECT/CT) neck acquisition is commonly used in both diagnostic [3] and post-therapeutic settings [4–7], there is still no other published report of RAI uptake in sinuses, especially in the maxillary or sphenoid ones. Furthermore, no cause other than chronic sinusitis or mucocele has evoked to explain sinus RAI uptake. We report here several cases of DTC patients showing RAI uptake in various sinuses (frontal, maxillary or sphenoid sinus), most of them being related to sinus mucosal thickening and one to a pathologically confirmed aspergilloma in a maxillary sinus. Each of these cases was clearly evidenced by SPECT/CT images.

## Case presentation

### Case 1

#### Sphenoid sinus

**Fig. 1:**
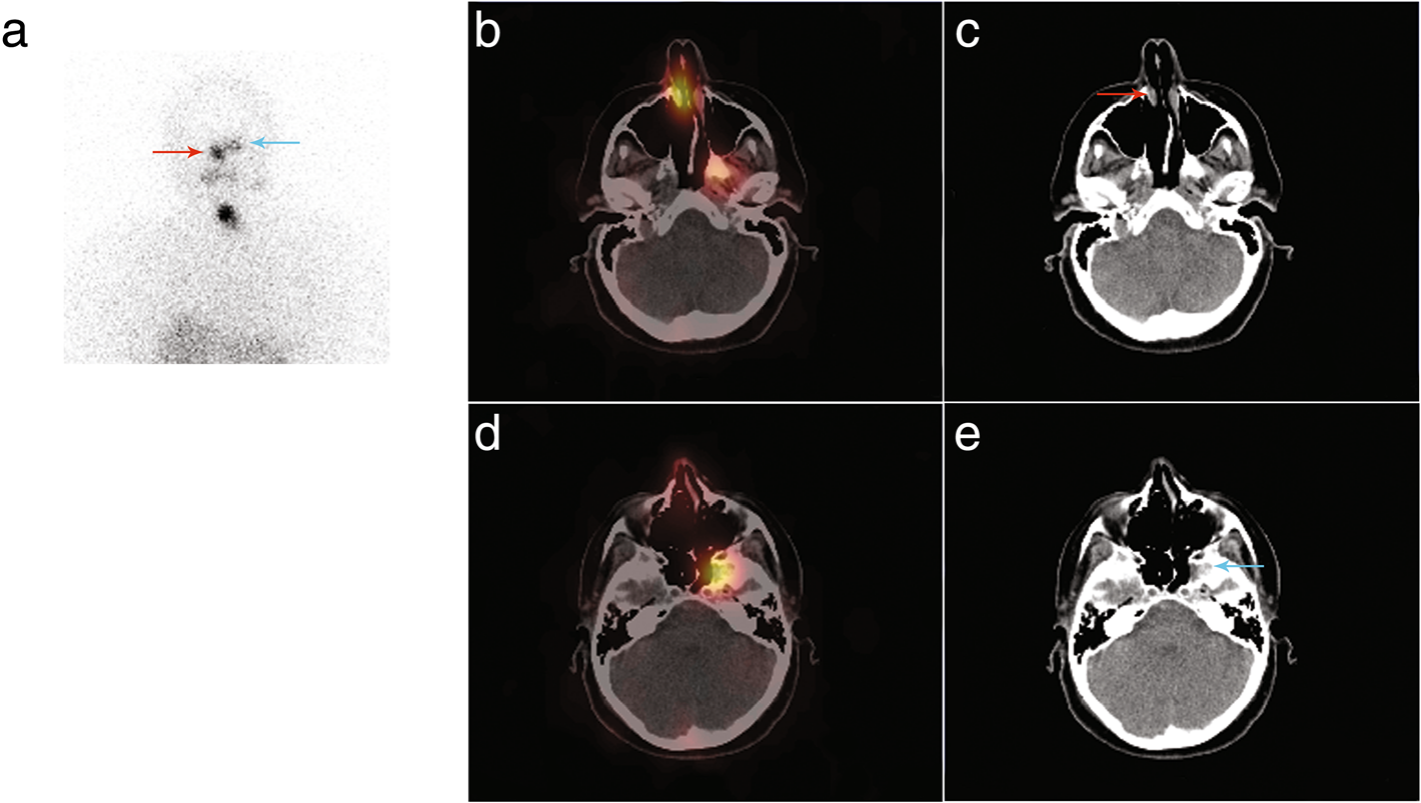
Planar acquisition (anterior view, Panel **a**), fused (Panel **d**) and CT (Panel **e**) images of SPECT/CT acquisition displaying RAI uptake in the left sphenoid sinus with mucosal thickening on CT scan slices (*blue arrow*). RAI uptake is also present in the nose (Panels **a**, **b** and **c**, *red arrow*)

A 61-year-old woman with papillary thyroid cancer (pT1b N0 Mx) was referred to our department in August 2010 for RAI ablation. Whole-body scan (WBS) with neck and thorax SPECT/CT (Fig. 1) was performed five days after administration of 4110 MBq (111 mCi) of RAI after recombinant human thyrotropin (rhTSH) stimulation. Stimulated thyroglobulin (Tg) was very low at 0.1 ng/ml without serum Tg antibodies (TgAb). WBS depicted an RAI focus in the nose related to nasal RAI excretion and a left-sided moderate uptake, also suggesting physiological excretion. Interestingly, SPECT/CT showed that the latter uptake was located in the sphenoid sinus with mucosal thickening. The patient had no symptoms. Sinus mucosal thickening had disappeared on the CT scan control in 2014 and she was disease-free at the last visit in October 2016.

### Case 2

#### Frontal sinus

**Fig. 2:**
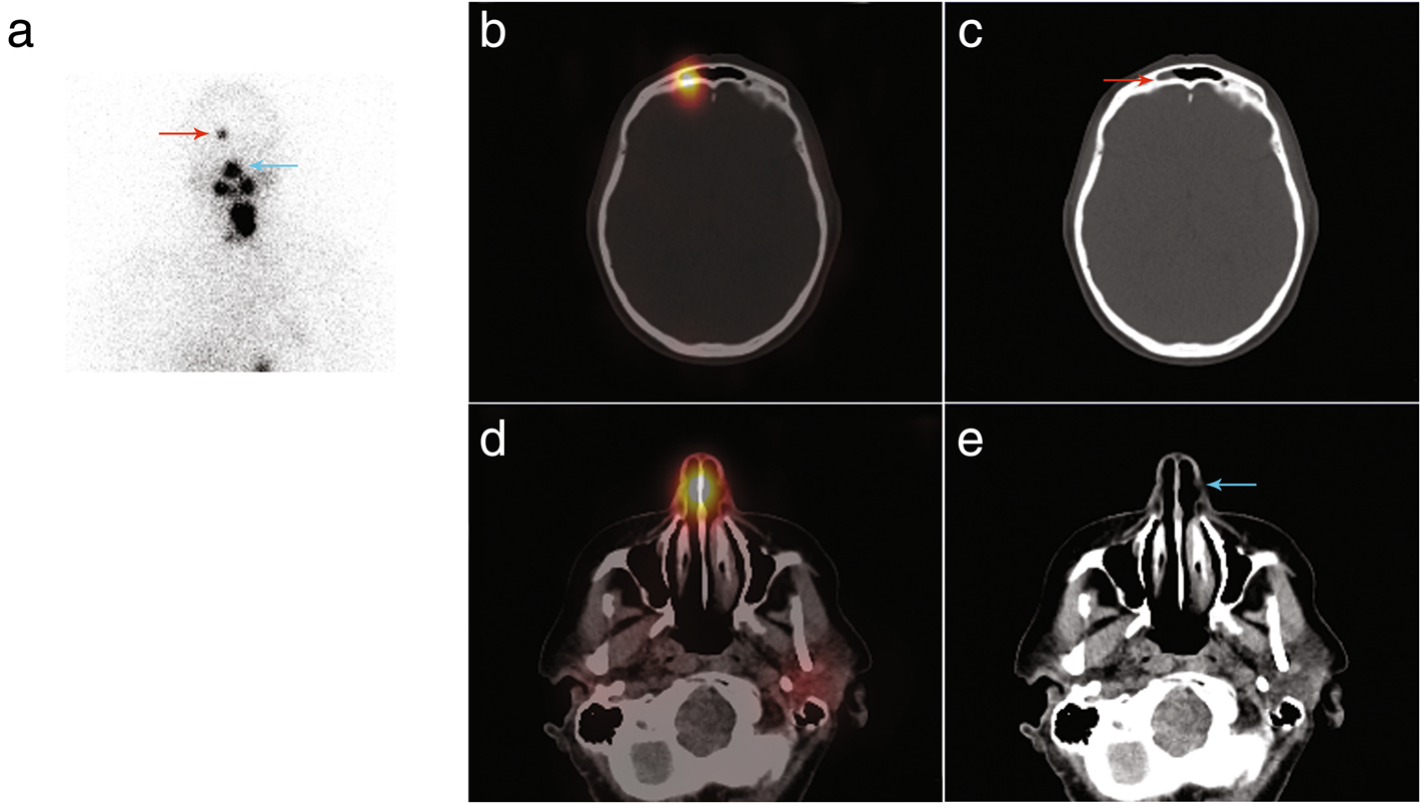
Planar acquisition (anterior view, Panel **a**), fused (Panel **b**) and CT (Panel **c**) images of SPECT/CT acquisition displaying RAI uptake in the right part of the frontal sinus with mucosal thickening on CT scan slices (*red arrow*). RAI uptake is also visible in the nose (Panels **a**, **d** and **e**, *blue arrow*)

A 51-year-old woman with a 15-mm papillary thyroid cancer with microscopic extrathyroid extension and central node involvement (pT3 N1a Mx) was referred for RAI ablation in January 2015. She was given 3570 MBq (96 mCi) of RAI after rhTSH. The stimulated Tg level was 0.3 ng/ml in the presence of serum TgAb. Five days after treatment, WBS evidenced focal RAI uptake in the right part of the skull. SPECT/CT acquisition ruled out bone metastasis and showed this uptake in the right frontal sinus to be associated with mucosal thickening (Fig. 2). The patient had no symptoms and was disease-free at the last visit in October 2016.

### Case 3

#### Frontal sinus

**Fig. 3:**
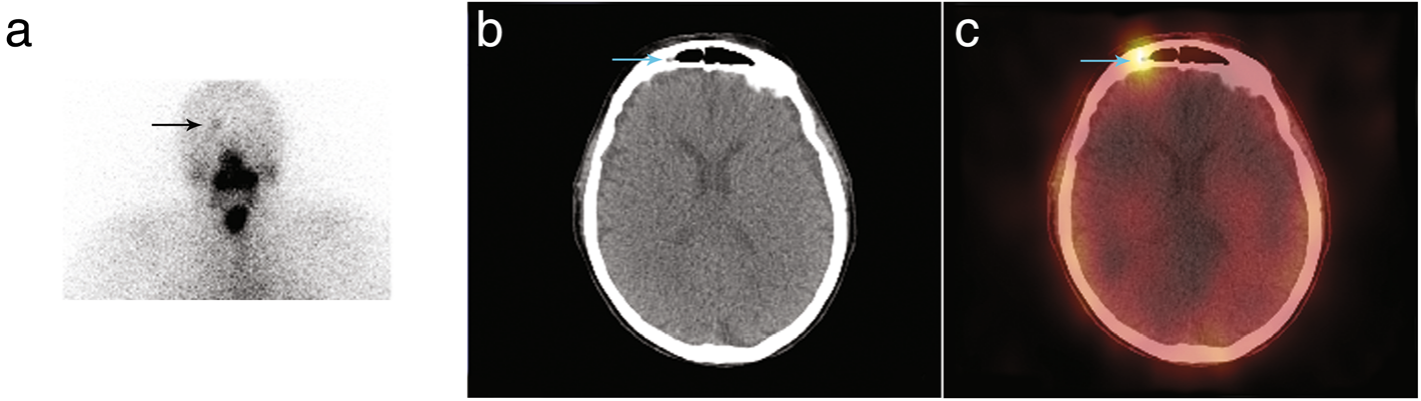
Planar acquisition (anterior view, Panel **a**), CT (Panel **b**) and fused (Panel **c**) images of SPECT/CT acquisition displaying a faint RAI uptake in the right part of the frontal sinus with mucosal thickening on CT scan slices (*blue arrow*)

**Fig. 4:**
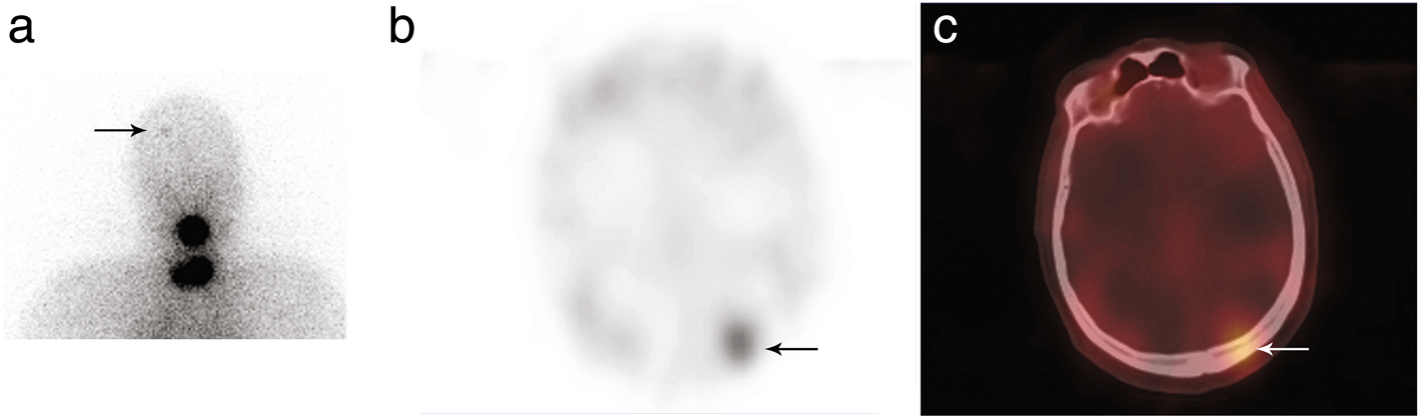
A 56-year-old male patient complained of back pain related to a lytic bone metastasis which led to the discovery of a follicular thyroid cancer. Post-ablation scintigraphy in March 2012 evidenced an intense RAI uptake in front of the spinal metastasis and other uptake in a rib and in the skull. Planar acquisition (posterior view, Panel **a**), SPECT (Panel **b**) and fused (Panel **c**) images of SPECT/CT acquisition displaying the faint RAI uptake in the left part of the skull are consistent with bone metastasis

An 81-year-old woman with a papillary thyroid cancer with lymph node involvement and microscopic extrathyroid extension (pT3 N1b Mx) was given 3681 MBq (99 mCi) of RAI in September 2015. The stimulated Tg level was 67 ng/ml. Post-ablation WBS depicted a faint RAI uptake in the anterior right part of the skull. SPECT/CT acquisition showed that this uptake was related to mucosal thickening in the frontal sinus (Fig. 3). For the sake of comparison, an example of a bone metastasis of the skull which was faintly RAI-avid and well depicted by SPECT/CT acquisition is presented in Fig. 4. Three months after RAI ablation, Tg level was at 22 ng/ml on levothyroxine. 18-Fluorodeoxyglucose positron emission tomography with computed tomography (18 F-FDG PET/CT) was performed in October 2016 and did not show any abnormal FDG uptake. We assumed that the patient had biochemical persistence of disease.

### Case 4

#### Maxillary sinus

**Fig. 5:**
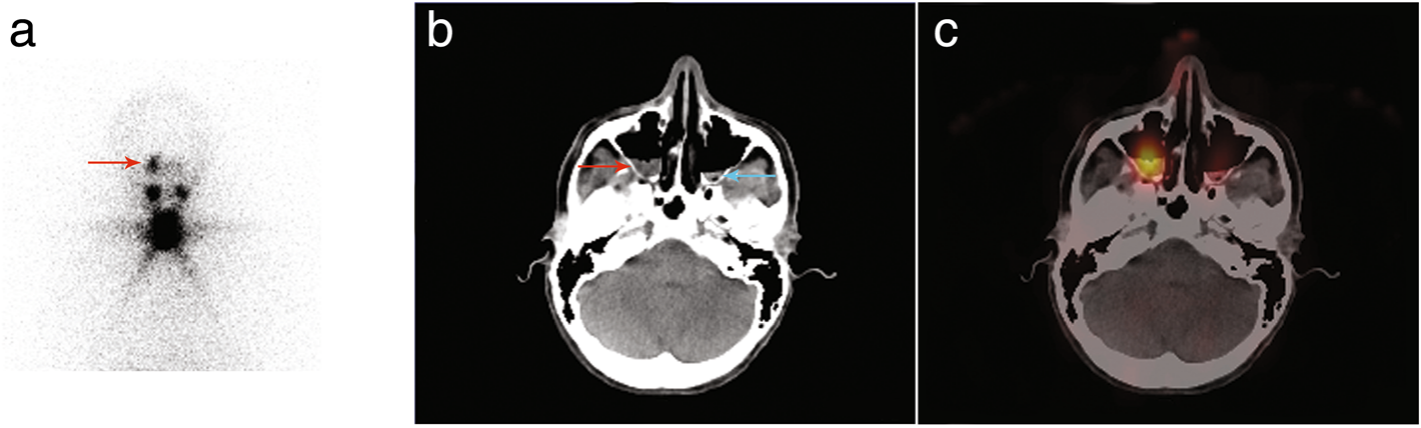
Planar acquisition (anterior view, Panel **a**), CT (Panel **b**) and fused (Panel **c**) images of SPECT/CT acquisition displaying RAI uptake in both the right maxillary sinus (*red arrow*) and the left one (*blue arrow*) with bilateral mucosal thickening on CT scan slices

A 49-year-old woman was referred for RAI ablation in April 2014 two months after total thyroidectomy for a 6-cm Hurtle cell carcinoma. She was given 3770 MBq (102 mCi) of RAI after rhTSH stimulation. The stimulated Tg level was low at 0.3 ng/ml without serum TgAb. WBS performed five days after treatment showed a right-sided moderate focus close to the nose suggesting physiologic excretion. On SPECT/CT this focus was clearly located in the right maxillary sinus, which showed mucosal thickening. SPECT/CT also revealed a faint uptake in the left maxillary sinus which was not seen on WBS (Fig. 5). The patient had no symptoms and was disease-free at the last visit in February 2016.

### Case 5

#### Maxillary sinus

**Fig. 6:**
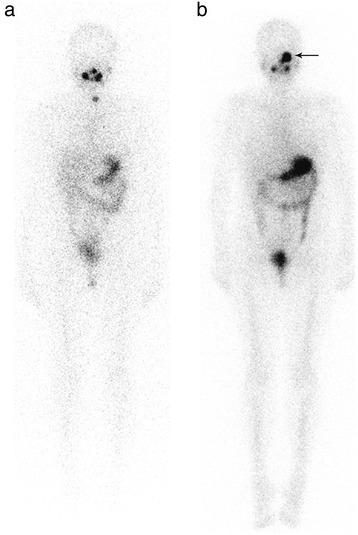
WBS after RAI ablation in August 2008 (Panel **a**) and WBS performed two days after the second RAI administration in June 2014 (Panel **b**) in the same female patient with increased serum Tg level. An intense focal RAI uptake was observed in the left part of the face in June 2014 (*arrow*) which was not present on the previous WBS

**Fig. 7:**
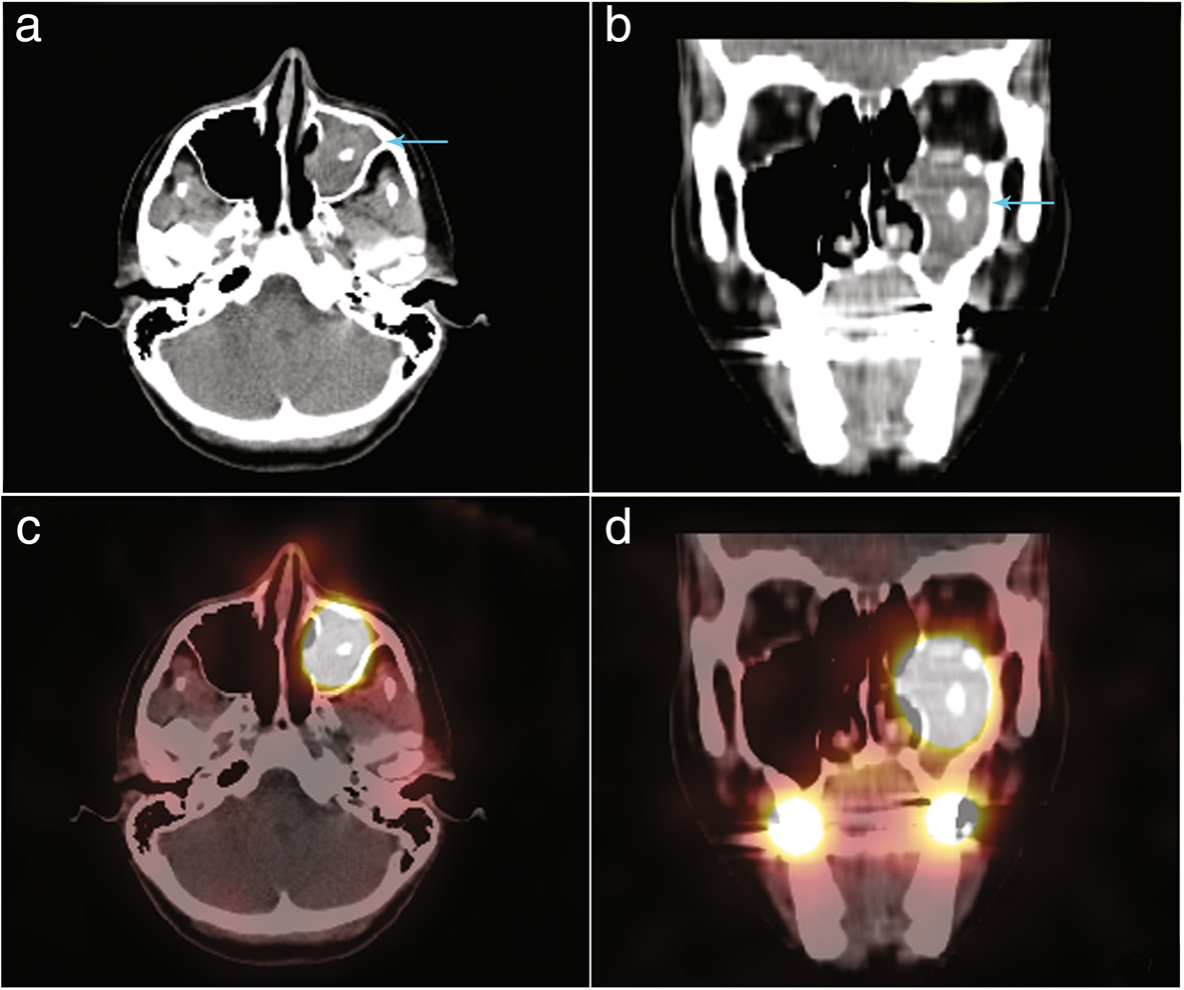
Complementary head and neck SPECT/CT was performed and confirmed that RAI uptake (fused images, Panels **c** & **d**) was located in the left maxillary sinus (hybrid CT scan images, Panels **a** & **b**, blue arrow). Hybrid CT scan evidenced a typical feature of aspergilloma with a dense soft-tissue mass including a calcification, suggesting a fungus ball

**Fig. 8:**
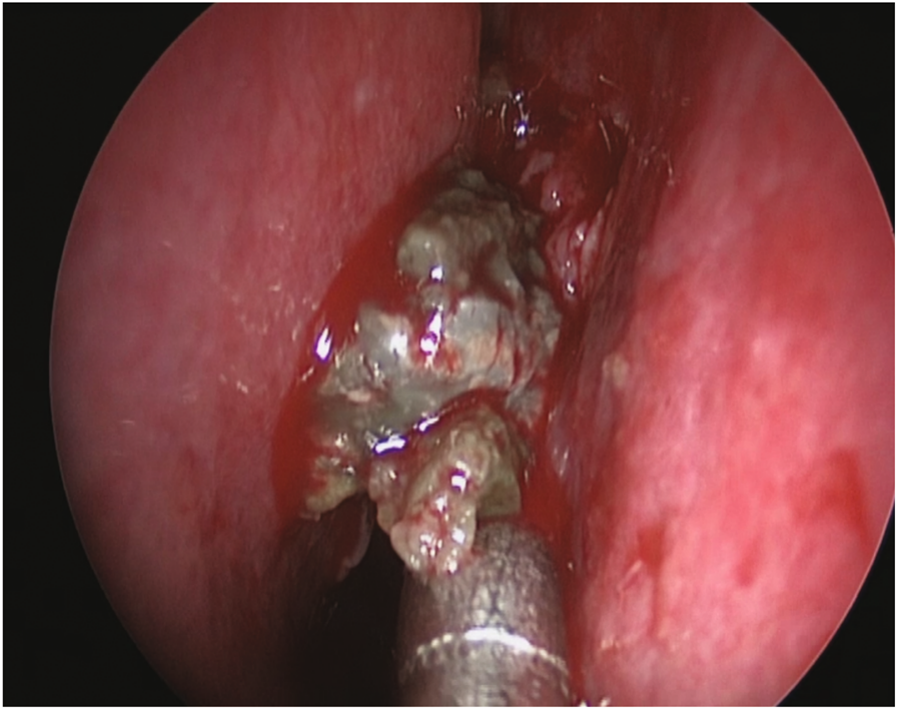
The patient underwent left sinus meatotomy endoscopically in February 2015 with removal of the fungus ball

A 38-year-old woman with a pT3 N1b M0 papillary thyroid cancer treated six years before by surgery and RAI ablation presented with an increased serum Tg level at 20 ng/ml on levothyroxine. Neck ultrasound examination did not show any abnormal lymph nodes. After a multidisciplinary staff meeting, she was given a second RAI treatment after rhTSH in June 2014, 1820 MBq (49 mCi). Stimulated Tg level was 61 ng/ml without serum TgAb. WBS performed two days after RAI administration depicted an intense focal uptake in the left part of the face which was not present on the post-ablation WBS in August 2008 (Fig. 6). Complementary head and neck SPECT/CT ruled out a bone lesion and showed that RAI uptake was located in the left maxillary sinus. Hybrid CT scan evidenced a typical feature of aspergilloma with a dense soft-tissue mass including a calcification, strongly suggesting a fungus ball (Fig. 7). The patient also complained of a stuffy nose. As WBS with SPECT/CT did not display malignant RAI foci, 18 F-FDG PET/CT was performed and evidenced FDG nodal uptake in the upper mediastinum. The patient was operated on in September 2014 with sternotomy to remove the FDG-avid lymph node. Pathology confirmed malignancy. She then underwent left sinus meatotomy endoscopically in February 2015 with removal of the fungus ball (Fig. 8). The stuffy nose complaint disappeared after surgery. At the last visit in April 2016, she was disease-free with a serum Tg level at 0.5 ng/ml.

## Discussion

This report describes five DTC patients in whom post-RAI SPECT/CT acquisition revealed benign RAI uptake in various sinuses (sphenoid, frontal or maxillary sinus) related to mucosal thickening in four patients and to aspergilloma in one. RAI sinus uptake has rarely been reported in the literature. Using planar imaging in 1994, Bakheet et al. reported frontal RAI uptake in a patient with chronic sinusitis [1]. In 1997, also with planar imaging, Matheja et al. described frontal RAI uptake in a patient with mucocele [2]. In a recent pictorial review using SPECT/CT [8], Glazer et al. listed unusual physiological RAI biodistributions at the head and neck level but no sinus uptake SPECT/CT image was presented. Our five case reports confirm the reality of sinus uptake and demonstrate that such benign uptake is not uncommon when SPECT/CT is routinely performed in combination with WBS.

In Case 3, despite the lack of histopathologic confirmation and the persistence of detectable Tg level without identifiable structural lesion, we concluded that sinus uptake was benign based on the pattern of RAI uptake on WBS with SPECT/CT.

In four patients without any symptom of sinusitis, incidental RAI uptake was due to sinus mucosal thickening. The mechanism of RAI sinus uptake remains elusive. As previously suggested [1], it might be due to the accumulation of inflammatory nasal secretions in the sinuses. As RAI is excreted in the salivary glands and the nose, moderate uptake in the maxillary and sphenoid sinuses can mimic physiological excretion and might remain unknown on WBS. Interestingly, RAI uptake was clearly related to aspergilloma in a maxillary sinus in the fifth patient. To our knowledge, this is the first such case to be reported although false-positive lung RAI uptake due to aspergillosis has already been documented [9–11]. Various fungi are known to be able to take up iodine-125 [12]. RAI uptake might be due to the presence of oxidants/antioxidants at sites of inflammation or to endotoxins or enzymes released by the fungus. However, the exact mechanism of such an accumulation in fungal cells remains unknown.

Thanks to its ability to localize RAI foci accurately on hybrid low-dose CT scans, SPECT/CT imaging has considerably altered the interpretation of planar scintigraphy in the last ten years. The improvement due to SPECT/CT is particularly significant when anatomical landmarks are obviously lacking as on post-RAI scintigraphy. Specifically, SPECT/CT was highly suggestive of aspergilloma on the hybrid CT scan without the need for further examinations in Case 5.

## Conclusions

In conclusion, false-positive RAI uptake in various sinuses related to mucosal thickening and aspergilloma is possible in patients with DTC and is well demonstrated by SPECT/CT images. Nuclear medicine physicians should be aware of such benign uptake when interpreting post-RAI scintigraphy.

## Abbreviations

18 F-FDG PET/CT: 18-Fluorodeoxyglucose positron emission tomography with computed tomography
DTC: Differentiated thyroid cancer
RAI: Radioiodine
rhTSH: Recombinant human thyrotropin
SPECT/CT: Single photon emission tomography with computed tomography
Tg: Thyroglobulin
TgAb: Thyroglobulin antibodies
WBS: Whole-body scan
